# Flexural Performance of Concrete Beams Reinforced with Continuous FRP Bars and Discrete Steel Fibers under Cyclic Loads

**DOI:** 10.3390/polym14071399

**Published:** 2022-03-30

**Authors:** Haitang Zhu, Chuanchuan Li, Shengzhao Cheng, Jiansong Yuan

**Affiliations:** 1 College of Civil Engineering, Henan University of Engineering, Zhengzhou 451191, China; htzhu@haue.edu.cn (H.Z.); yuanjiansong@hotmail.com (J.Y.); 2 School of Water Conservancy Engineering, Zhengzhou University, Zhengzhou 450001, China; 3 China Construction Seventh Engineering Division Co., Ltd., Zhengzhou 450004, China; chengshengzhao@aliyun.com

**Keywords:** BFRP bar, flexural performance, cyclic loading, reinforcement ratio, deformability

## Abstract

This research investigated the flexural behavior of high-strength concrete beams reinforced with continuous basalt fiber-reinforced polymer (BFRP) bars and discrete steel fibers. Five concrete beams with the dimensions of 150 × 300 × 2100 mm^3^ were constructed and tested to failure under four-point bending cyclic loading. The specimens consisted of four BFRP-reinforced concrete beams with various reinforcement ratios (*ρ*_f_), namely, 0.56%, 0.77%, 1.15%, and 1.65%, and one conventional steel-reinforced concrete beam for comparison purposes. The cracking behavior, failure modes, load-deflection behavior, residual deformation, and stiffness degradation of the beams were studied. Additionally, a deformation-based approach was used to analyze the deformability of the beams. The results show that an increase in the *ρ*_f_ effectively restrained the crack widths, deflections, and residual deformation while also enhancing the flexural bearing capacity of the beams. In comparison to the first displacement cycle, the bearing capacity dropped by 10% on average in the third cycle. The stiffness exhibited a fast to slow degradation trend until failure. The residual stiffnesses were higher in beams with a higher *ρ*_f_. The over-reinforced beams had superior deformability than the under-reinforced beams, according to the deformability factors.

## 1. Introduction

Reduced load-bearing capacity and durability caused by steel corrosion in conventional reinforced concrete (RC) structures have become major problems in engineering today. Engineering practice shows that when exposed to aggressive environments, corrosion of steel reinforcement accelerates and the durability and service life of RC structures is severely reduced [1,2,3,4,5]. Fiber-reinforced polymer (FRP) bars are increasingly being used to replace steel bars in conventional RC members, owing to their excellent corrosion resistance. In addition, FRP bars are electrically insulating and possess a high tensile strength and strength-to-weight ratio compared to steel bars [6,7]. They have been widely applied in engineering structures in recent decades, especially in aggressive environments [8,9,10,11].

There are several types of FRP materials used as reinforcement in concrete structures, such as aramid FRP (AFRP), carbon FRP (CFRP), glass FRP (GFRP), and basalt FRP (BFRP). The most commonly used types are CFRP, GFRP, and AFRP [12,13]. However, according to Sim’s [14] research findings, the performance of GRRP and AFRP could be significantly affected by the alkaline environment within the concrete, whereas the CFRP bars are too expensive to realize large-scale application in civil engineering structures [15]. Advances in FRP technology resulted in increasing demand to introduce new types of fibers. Under these circumstances, a new type of FRP, basalt FRP (BFRP), with superior performance and broad application potential emerged and attracted the researchers’ and manufacturers’ interests in recent years [12,16,17,18,19,20,21]. BFRP is a new environmentally friendly reinforcing material with a high performance–price ratio, high fire resistance [22], excellent freeze–thaw performance [23], and ease of manufacture. The BFRP composites showed potential for application in various areas such as national defense, aerospace, civil construction, transportation infrastructure, energy infrastructure, fire protection, automobiles, water conservation, and hydropower [24]. According to a previous study, BFRP has a higher strength and elastic modulus than GFRP, as well as greater chemical stability [22]. The research results of Wei [25] and Lee [26] revealed, respectively, that BFRP bars have superior function in acidic and alkaline conditions in comparison to GFRP bars. Additionally, it also has a wider range of working temperatures and much lower costs than CFRP [20,22]. Furthermore, the current FRP design codes, such as CSA S6-06 [27] and ACI 440.1R-15 [28], make no suggestions for BFRP-RC structural elements [20,29]. The findings of current research are insufficient to provide a design basis for BFRP-RC elements. Therefore, more research is needed to investigate factors that significantly affect the design, constructability, and performance of BFRP in RC elements [30].

However, the linear elastic stress–strain nature of FRP bars means that the design of FRP-RC members requires special attention to be paid to the serviceability issues, such as larger deflections, wider cracks widths, and reduced ductility [31,32,33]. Moreover, the low elastic modulus of FRP bars causes an abrupt drop in stiffness when the concrete cracks, which seriously affects the post-cracking performance of structures [34,35]. Therefore, the failure mode of concrete crushing rather than FRP bar rupture was recommended by ACI440-1R-15 [28] for FRP-RC members. Previous studies also prove that the inclusion of randomly distributed fibers could effectively restrain the deflections and crack widths and improve the deformability and flexural capacity of FRP-RC beams [12,16,36,37,38]. Besides, the bridging effect of fibers helps to control the compression failure of fiber-reinforced concrete (FRC) beams, allowing the high strength of FRP bars to be effectively utilized [12]. Gao [39] and Li [40] suggested the maximum volume ratio of steel fiber (SF) to be 1.5% and 1.0% for concrete beams. In addition, studies by Mydin [41] found that the inclusion of SF at approximately 0.75–1.0% content in volume within a concrete mixture helped improve its flexural strength and ductility.

Some recent studies have concentrated on the flexural performance of BFRP-RC beams subjected to four-point bending static loads [12,20,29,30,36,40]. Ovitigala [30] found that all the tested beams had failed by the top concrete crushing. A higher reinforcement ratio (*ρ*_f_) has a better effect on reducing the deflection than on increasing the ultimate strength. The strain compatibility equation suggested by ACI was conservative in predicting both the ultimate flexural strain in the BFRP bars and the ultimate moment capacity. Elgabbas’s [20] experimental results yielded an average bond-dependent coefficient of 0.83 for BFRP bars, which is lower than the 1.0 recommended by Canadian standards for ribbed FRP bars. Hua’s [29] test results show that with the *ρ*_f_ increasing, the failure mode of the specimen converted from tensile failure to balance failure, and ultimately to compressive failure. The flexural capacity, crack width, and deflection of the BFRP-RC beams are much higher than that of a steel-RC beam. Increasing the *ρ*_f_ can increase the flexural stiffness of the beams, which consequently enhances the flexural capacity and reduces the strain, deflection, and crack width of the beams. The experimental results of Farid Abed [12] demonstrate that the flexural capacities of BFRP-RC beams were slightly underestimated by ACI440.1R-15 [28], while reasonable predictions for the cracking moments were observed. The use of high-strength concrete enhanced the cracking and ultimate moments of all BFRP-RC beams by 10% and 16%, respectively, when compared to normal strength concrete. Furthermore, the average bond-dependent coefficient value for BFRP-RC beams was found to be 0.70, which is significantly lower than the conservative value suggested by the ACI guidelines. Zhu [36,40] studied the flexural behavior of fiber-reinforced high-strength concrete beams reinforced with BFRP bars under four-point bending tests. The results show that a higher *ρ*_f_ results in improved flexural performance, including higher flexural strength, post-cracking stiffness and ductility, and smaller crack width.

In practical engineering, many structures, such as highway and railway bridges, airport pavements, and offshore elements, are continuously subjected to cyclic loads during their service life, such as traffic loads, wind loads, and seismic loads [42,43]. To date, studies regarding BFRP-RC structures reinforced with or without fibers under monotonic loading have been widely conducted and published [12,13,19,20,24,36]. However, studies on these structures subjected to cyclic loading are still limited. Therefore, the influence of the dynamic load on the FRP-RC structures should be investigated to eliminate the negative impact of the dynamic load on the structural design and construction.

The objective of this research was to study the flexural behavior of steel fiber-reinforced concrete (SFRC) beams reinforced with different ratios of BFRP bars under cyclic loading. A four-point bending test was carried out to evaluate the flexural behavior of these beams on cracking pattern, failure modes, load-deflection behavior, and stiffness degradation under cyclic loading. In addition, a deformation-based approach, which is suitable for FRP-RC members, was employed to assess the deformability of BFRP-SFRC beams.

## 2. Experimental Investigation

### 2.1. Materials and Mix Proportion

**Figure 1:**
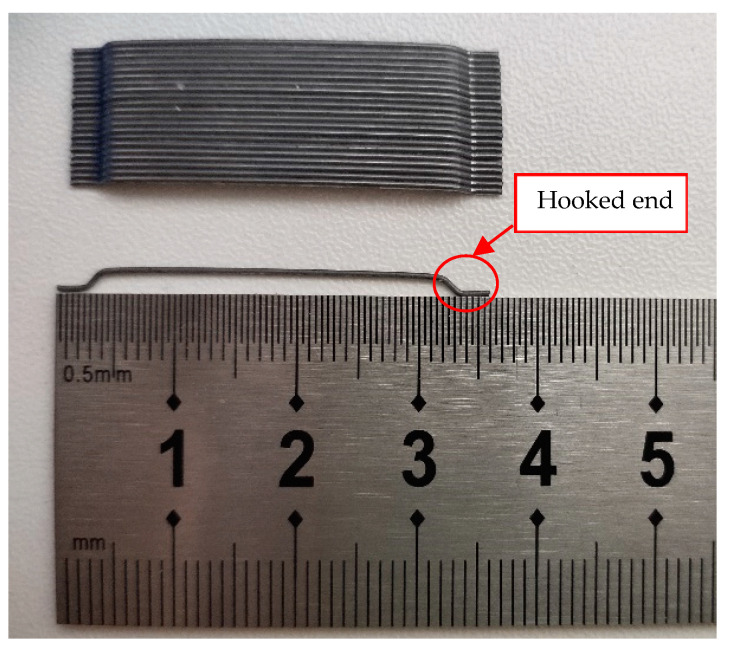
Dimensions of SF.

**Figure 2:**
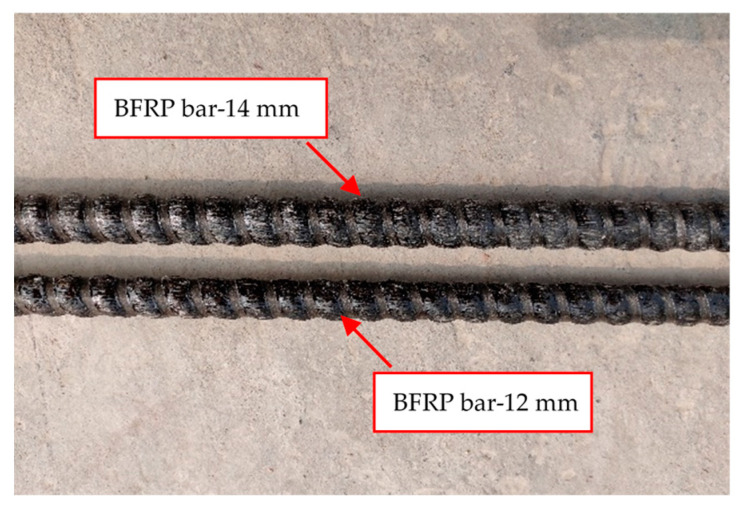
BFRP bars.

The composition and mix proportion (by weight) of the concrete are given in Table 1. Ordinary Portland cement of 42.5 grades (P.O 42.5), which was produced in Tianrui Cement Co., Ltd., Zhengzhou, China, was used as the cementitious material in the mix. Natural river sand with a maximum size of 5 mm was used as fine aggregate. Gravel within a size range of 5–20 mm was used as coarse aggregate. Polycarboxylic acid water reducer produced by Sobute New Materials Co., Ltd., Nanjing, China, was used for good workability of the concrete. 0.55 mm diameter SF with a total length of 35 mm were incorporated at a volume ratio of 1%. SF was manufactured by Bekaert, Shanghai, China. The dimension and detailed properties of the SF are shown in Figure 1 and Table 2, respectively. The 28 day average cubic compressive strength and splitting tensile strength of SFRC was 72.5 MPa and 6.30 MPa, respectively. BFPR bars with diameters of 12 mm and 14 mm (see Figure 2) and 14 mm diameter steel bars were used as longitudinal reinforcements. Steel bars with diameters of 10 mm and 6 mm were used as stirrup and erection bars, respectively. BFPR bars were manufactured by GMV New Material Technology Development Co., Ltd., Nanjing, China. Figure 2 shows the surface features of BFRP bars. Table 3 shows the mechanical properties of the reinforcing bars.

### 2.2. Test Specimens

**Figure 3:**
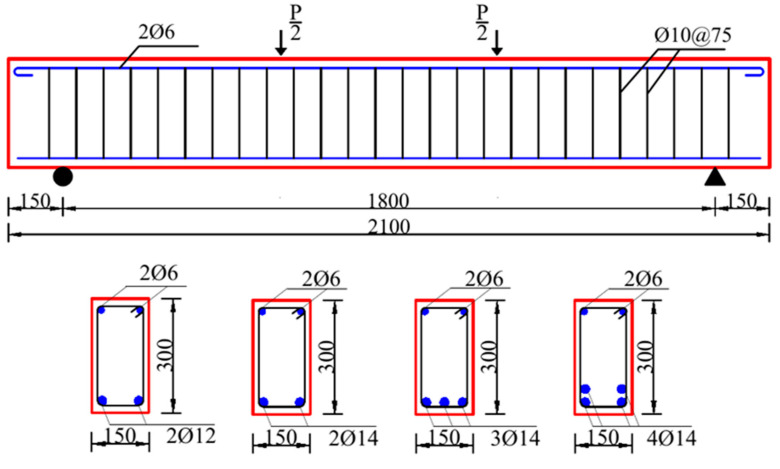
Specimen geometry and reinforcement.

A total of five beams were designed, four of which were reinforced with different amounts of BFRP bars and one with steel bars. The beams had the same dimensions of 150 × 300 × 2100 mm^3^ with a concrete cover of 15 mm. The longitudinal reinforcements were 12 mm and 14 mm diameter BFRP bars and 14 mm diameter steel bars. Steel bars with a 10 mm diameter were used as stirrups at 75 mm spacing to prevent shear failure. The cross-sectional dimensions and reinforcement details are illustrated in Figure 3. Four different *ρ*_f_ values were selected in this test: 0.56% (2Φ12), 0.77% (2Φ14), 1.15% (3Φ14), and 1.65% (4Φ14). Accordingly, each beam was assigned a unique identification, i.e., B56, B77, B115, B165, and S77, in which the letters B and S denote the BFRP and steel reinforcement, respectively, and the number represents the *ρ*_f_ in ‱.

### 2.3. Test Setup, Instrumentation and Loading Procedure

**Figure 4:**
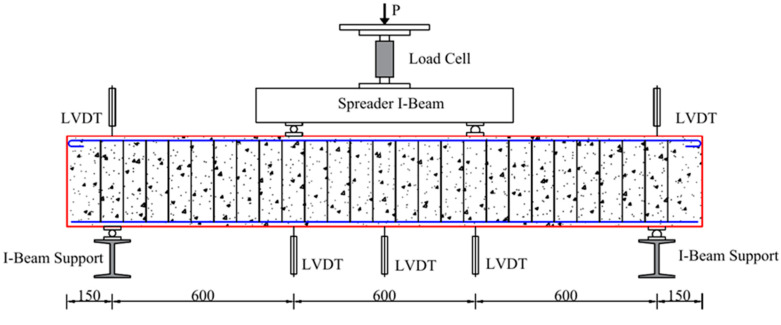
Test setup and instrumentation.

**Figure 5:**
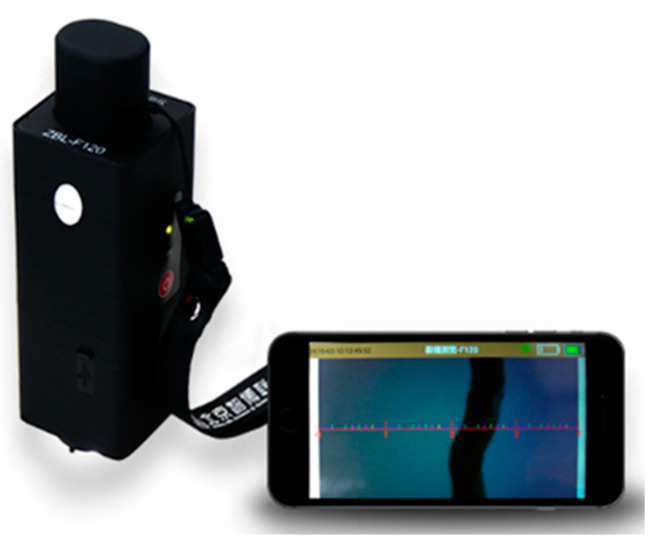
Crack width detector.

The test setup and instrumentation are illustrated in Figure 4. The beams were all simply supported and tested to failure under four-point bending cyclic loading. Each beam had a clear span of 1800 mm and a 150 mm extension outside each support. The constant moment region had a length of 600 mm, which was the same as the shear region. A hydraulic actuator with a load capacity of 500 kN was utilized to exert the load, which was connected to a steel I-beam to transfer the load to the loading points. Five linear variable differential transformer (LVDT) sensors were employed to record the deformation of the beams. Crack width detectors (see Figure 5) were used to measure the crack widths.

**Figure 6:**
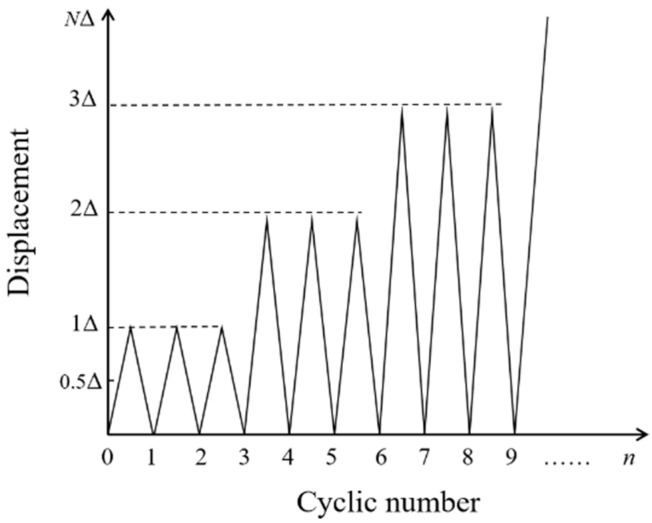
Scheme of loading.

The cyclic loading was executed under displacement control with a target loading–unloading rate of 2 mm/min. The displacement increment Δ was set at 6 mm. Three loading–unloading cycles were applied to the specimens at each displacement level (Δ, 2Δ, 3Δ…), after which the displacement increased to a higher level, as Figure 6 shows. When the bearing load of beams dropped below 80% of the ultimate load, the test stopped.

## 3. Results and Discussion

In this section, the cracking behavior, failure modes, load-deflection behavior, stiffness degradation, and ductility and deformability of the beams are discussed. The flexural test results are summarized in Table 4.

### 3.1. Cracking Behavior and Failure Modes

**Figure 7:**
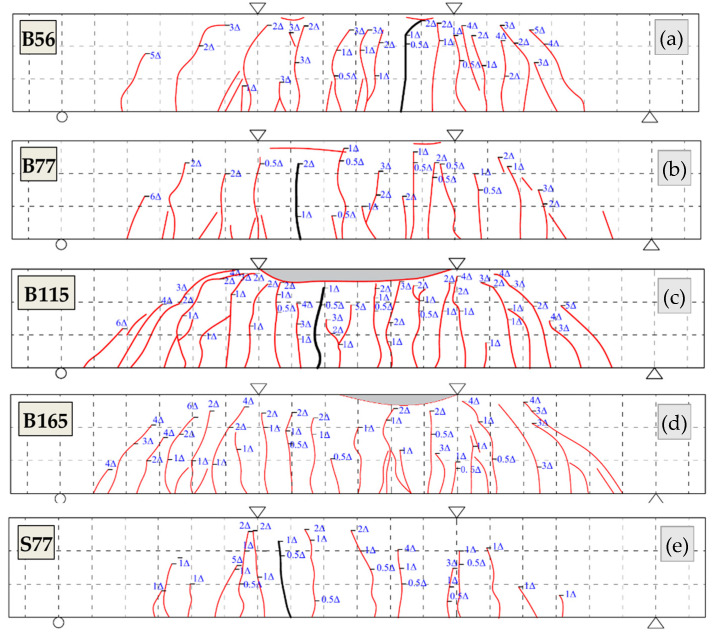
Crack patterns: (**a**) B56; (**b**) B77; (**c**) B115; (**d**) B165; (**e**) S77.

The initial cracks all appeared in the pure bending zone of the beams. Compared with beam B56, the *ρ*_f_ of beams B77, B115, and B165 increased 38%, 105%, and 195%, respectively, while the crack loads increased only 5%, 14%, and 24%, respectively, which indicates that the *ρ*_f_ increasing had a minor influence on the crack load. Beam S77 had the largest crack load due to the high stiffness of steel reinforcement. The crack distribution diagram is presented in Figure 7. When the displacement reached 0.5Δ, the maximum crack widths reached 0.20 mm, 0.16 mm, 0.14 mm, 0.10 mm, and 0.10 mm for beams B56, B77, B115, B165, and S77 at the corresponding loads of 57.0 kN, 60.3 kN, 63.8 kN, 69.8 kN, and 84.4 kN, respectively. When the displacement level was increased to 1Δ, the crack widths and number of cracks both increased. New cracks initiated and propagated at the shear zone of beams. However, the crack widths hardly changed after the second and third cycles. When the displacement level was increased to 2Δ, cracks in the pure bending zone had developed completely and propagated to a considerable height (over 70% of the beam depth). Cracks in the shear zone developed rapidly during this stage. With further loading, new cracks initiated and propagated only in the shear zone. The cracks widths kept increasing with the increasing displacement level until the beam failed. Figure 7 shows that the number of cracks in beams B115 and B165 is more than that in beams B56 and B77, thereby resulting in smaller crack spacing.

**Figure 8:**
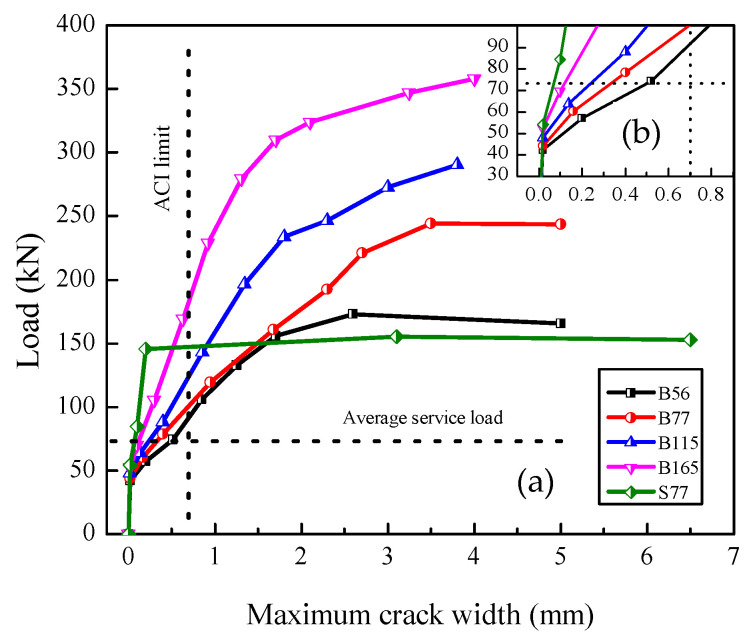
(**a**) Load-maximum crack width curves; (**b**) Load-maximum crack width under service load.

Figure 8 illustrates the load–maximum crack widths curves of the beams. In BFRP-RC beams, the crack widths reduced with the *ρ*_f_ increasing under the same load level. At the average service load (30% of the average ultimate load), the maximum crack widths decreased 34%, 52%, and 76% with the *ρ*_f_ increased from 0.56% to 0.77%, 1.15%, and 1.65%, respectively. In beam S77, the crack widths were small under the initial loading due to the high elastic modulus of steel bars. After yielding, the crack widths increased dramatically and were much wider than that of the BFRP-SFRC beams. ACI440.1R-15 [28] suggests 0.7 mm as the maximum crack width limit for FRP-RC flexural structures. The crack widths under the service load (30% of the ultimate load) in Table 4 and under the average service load in Figure 8 are all within the ACI crack width limit for all the beams. For beams that failed by concrete crushing, B165 had a larger crack width under the ultimate load but a smaller one under the service load compared to that of B115, which indicates that the increase in the *ρ*_f_ improves the serviceability of beams.

**Figure 9:**
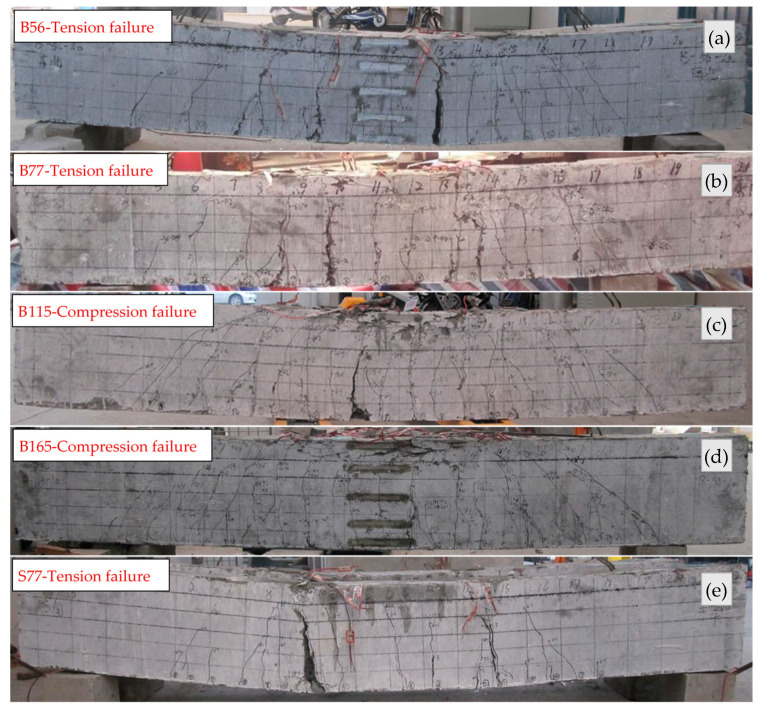
Failure modes: (**a**) B56; (**b**) B77; (**c**) B115; (**d**) B165; (**e**) S77.

During the test, the beams exhibited two typical failure modes: tension failure and compression failure, as Figure 9 shows. Beams B56 and B77 exhibited tension failure and the BFRP bars ruptured ‘/bars ruptured. When the load of beam B165 dropped below 80% of the maximum load, the BFRP bars were still working. The deformation of beam B165 could almost be restored after unloading.

### 3.2. Load-Deflection Curves and Residual Deformation

**Figure 10:**
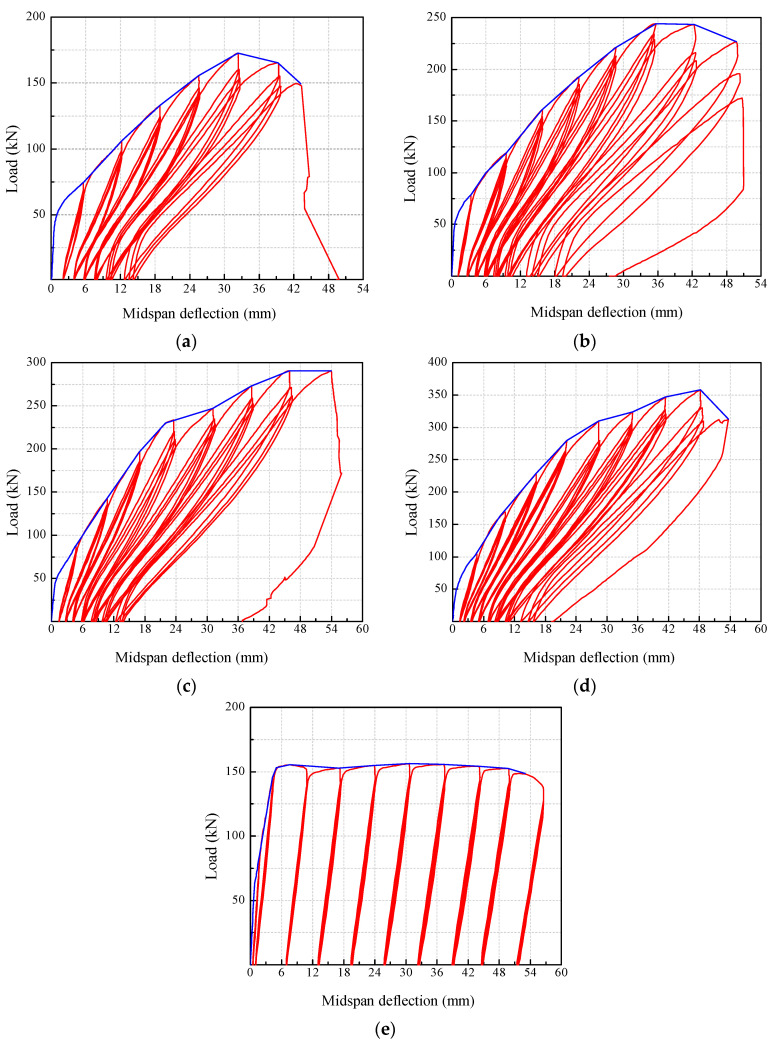
Load-deflection curves: (**a**) B56; (**b**) B77; (**c**) B115; (**d**) B165; (**e**) S77.

Figure 10 presents the load-deflection curves of the beams. The enclosed area of the loading–unloading curves of the BFRP-SFRC beams decreased gradually with the *ρ*_f_ increasing. This indicates that the stiffness of the beams was enhanced by increasing the amount of BFRP bars. As a result, the beams with a higher *ρ*_f_ reached a higher displacement level and experienced more loading–unloading cycles. For each beam, the loading–unloading curves of the second and third cycle almost coincided when the deflection was at a lower level. As the deflection grew larger, the spacing between the loading–unloading curves of the second and third cycle grew gradually larger. This demonstrates that the stiffness of the beams degraded with the increase in displacement and load cycles. In beam S77, the deflection increased linearly with the load at the initial stage. After yielding, the deflection increased rapidly while the bearing load remained unchanged. The enclosed area by the loading–unloading curve was similar at each displacement level, which indicates that the stiffness of the beam reduced significantly after yielding.

Under the same displacement level, the bearing capacity of the beams slightly decreased with the increase of load cycles due to damage accumulation inside the beam. While at the late loading stage, the decrement of flexural bearing capacity increased significantly, indicating that the beam was on the verge of destruction. Table 5 summarizes the flexural bearing capacity degradation factor at each displacement level for all the beams. The flexural bearing capacity degradation factor was the ratio of bearing capacity at the third cycle to that at the first cycle at each displacement level [44]. It can be seen that the majority of the factors were all above 90% until the beams failed. The average degradation factor of beams failed by compression failure is only 1% larger than that of beams failed by tension failure, which indicates that the BFRP *ρ*_f_ had a negligible effect on the bearing capacity degradation of the beams.

**Figure 11:**
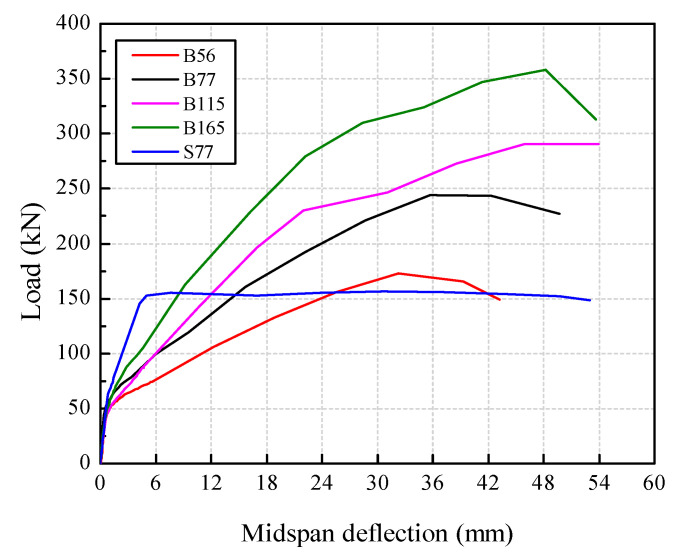
Skeleton curves.

Figure 11 presents the skeleton curves of the beams. The beams with higher a *ρ*_f_ obtained higher ultimate flexural capacities. The ultimate flexural bearing capacity of the beams B77, B115, and B165 increased by 48%, 76%, and 117%, respectively, compared with that of B56, which were much larger than the increments in crack load. The deflections of the BFRP-SFRC beams grew non-linearly with the load increasing but decreased significantly with the *ρ*_f_ increasing. At the service load (30% of the ultimate load), the deflections of the over-reinforced beams B115 and B165 were 4.73 mm, and 4.80 mm, respectively, which were all within the limitation in ACI (*l*/180, *l* is the clear span of the beam). With the *ρ*_f_ increased from 1.15% to 1.65%, the deflection increased 5% under the ultimate load but 1.4% under the service load. This demonstrates that the increase in the *ρ*_f_ had a stronger influence on the deflections under the serviceability limit state than the ultimate limit state, which is consistent with the conclusion on the crack width. Before yielding, the deflection of beam S77 increased linearly with the load and was smaller than that of the BFRP-SFRC beams. Afterward, the deflection increased continuously with the load sustained at approximately 150 kN, which was far larger compared to that of the BFRP-SFRC beams.

**Figure 12:**
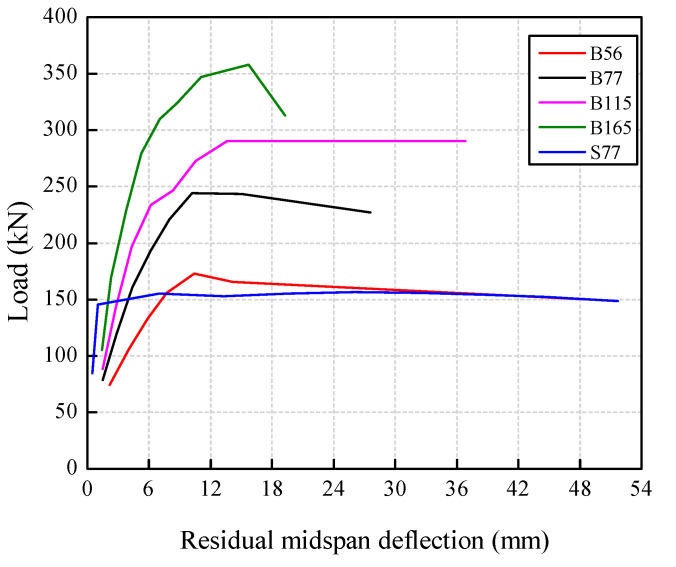
Residual deflection.

The load–residual deflection curves are presented in Figure 12. Similar to the skeleton curves, the residual deflection decreased significantly with the *ρ*_f_. Considering the linear elastic characteristic of BFRP bars, the deformation of the BFRP-SFRC beams can be partly restored after unloading at each displacement level. Therefore, the residual deflection of beams B56, B77, B115, and B165 increased at a relatively lower rate in the initial loading stage. With the load cycles and displacement levels increasing, the internal damage of concrete gradually accumulated, resulting in a faster increase in residual deflection. Large plastic deformation appeared after steel bar yielding in beam S77, resulting in a rapid increase in residual deformation, which was larger than that of the BFRP-SFRC beams. At the failure stage of the test, the break of BFRP bars in beams B56, B77, and B115 resulted in a much larger ultimate residual deflection than beam B165. Since beam B165 was failed by concrete crushing, the ultimate deflection kept decreasing after unloading due to the linear elastic stress–strain relationship of the BFRP bars. Therefore, it can be concluded that the residual deformation of BFRP-SFRC beams with a higher *ρ*_f_ can be effectively controlled under cyclic loading.

### 3.3. Stiffness Degradation

**Figure 13:**
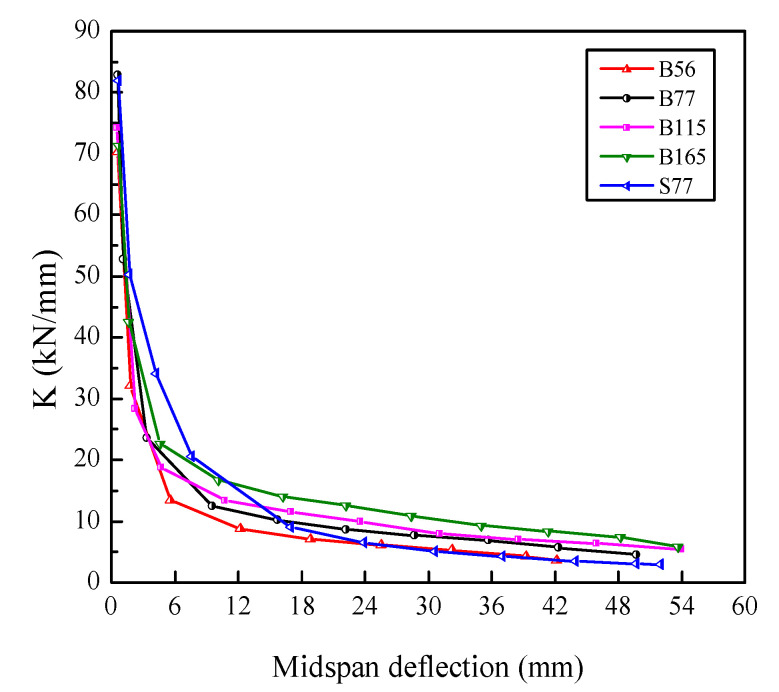
Equivalent stiffness degradation.

In this study, equivalent stiffness was utilized to study stiffness degradation. The equivalent stiffness (K) was defined as the secant of the skeleton curve, namely, the ratio of the peak load to the corresponding deflection at the first cycle of each displacement level. Figure 13 illustrates the stiffness degradation of the beams. The stiffness of the beams had a similar degradation trend. At the initial loading stage, all beams had high stiffnesses and stiffness degradation rates. This is mainly due to the continuous initiation and propagation of new cracks in the early stage of loading, which leads to damage accumulation in concrete. With further loading, the stiffness degradation rate decreased significantly. This can be attributed to the bridging effect of SF, which restrained the deformation of the beams during this stage. With a further increase in the displacement, the stiffness degraded at a lower rate until the beams failed. It is noticeable that the beams with a higher *ρ*_f_ had higher residual stiffnesses. In beam S77, the stiffness degraded more slowly in the early stage of loading and faster in the later stages compared with the BFRP-SFRC beams.

### 3.4. Ductility and Deformability

Ductility is one of the most important structural design criteria, and is defined as the ability of a beam to maintain inelastic deformation without losing its load-bearing capacity before failure [31]. Structures with good ductility can exhibit early warning before failure, whereas for brittle structures, little or no warning is presented before failure. In traditional RC structures, the ductility index is defined as the ratio of ultimate deflection to yield deflection. However, this definition is no longer applicable to FRP-RC members, since FRP bars present linear elasticity until failure [37,45]. Accordingly, several methods were suggested to evaluate the ductility for FRP-RC structures. Naaman and Jeong [46] and Jaeger et al. [47] first introduced the energy-based method and the deformation-based method, respectively, to determine the ductility index for FRP-RC beams. However, the deformation-based method was reported to be more applicable than the energy-based method when considering the contribution of SF on the ductility of FRP-RC beams [46,48]. Consequently, in this study, the deformation concept was adopted to assess the ductility of BFRP-SFRC beams. The deformability factor J is defined as follows:

J = C_S_ × C_C_ (1)

C_S_ = M_u_/M_ε = 0.001_ (2)

C_C_ = ϕ_u_/ϕ_ε = 0.001_ (3)

where C_S_ = the moment coefficient; C_C_ = the curvature coefficient; M_u_ = ultimate Moment; ϕ_u_ = ultimate curvature; M_ε = 0.001_ = moment at a concrete compressive strain of 0.001; and ϕ_ε = 0.001_ = curvature at a concrete compressive strain of 0.001.

The calculation results are shown in Table 6. It is suggested by Jaeger et al. [47] and CSA-S6-06 [27] that the deformability factor for FRP-RC beams should not be less than 4. The deformability factor J varied from 6.54 to 10.68 in Table 6. This suggests that BFRP-SFRC beams have excellent deformability. For under-reinforced beams (i.e., B56 and B77), the high compressive strength and ultimate strain of SFRC in the compression zone were not fully developed due to the rupture of the BFRP bars. Therefore, the deformability factor was lower. For over-reinforced beams (i.e., B115 and B165), the high compressive strength and ultimate strain of SFRC were fully exploited before the BFRP bars ruptured. The values of factor J were larger than that of under-reinforced beams but slightly reduced with the *ρ*_f_ further increased, which indicates that a further increase in BFRP *ρ*_f_ did not improve the deformability of over-reinforced beams. Therefore, to obtain better deformability, the *ρ*_f_ of over-reinforced beams should be conservative.

## 4. Conclusions

The objective of this work was to investigate the flexural behavior of BFRP-SFRC beams under four-point bending cyclic loads. The following conclusions were derived from the above discussions on the BFRP-SFRC beams:

(1) The increase in reinforcement ratio led to a notable decrease in crack widths. The enclosed area by the loading–unloading curves gets smaller with the increase in the reinforcement ratio. This reveals that the stiffness of the beams was strengthened by the increase in the reinforcement ratio. The beams with a higher reinforcement ratio could bear larger displacement levels and more loading–unloading cycles.
(2) The BFRP-SFRC beams exhibited good serviceability with the increase in the reinforcement ratio for over-reinforced beams. The crack widths and deflections of all the beams at service load were all within the ACI limit.
(3) With the increase in the displacement level and load cycles, the stiffness of the beams gradually reduced. The midspan deflections and residual deflections were effectively restrained by increasing the amount of BFRP reinforcement.
(4) The bearing capacity of the beams slightly degraded with the load cycles. The flexural bearing capacity degradation factors were mostly above 90% before failure and were negligibly influenced by the reinforcement ratio under different displacement levels. The ultimate flexural capacities of the beams were significantly improved by increasing the BFRP reinforcement ratio.
(5) The stiffness of the beams degraded rapidly in the early stage of loading and then slowly until failure. The beams with higher reinforcement ratios had larger residual stiffnesses. The stiffness of the beams reinforced with steel bars degraded more slowly in the initial loading, but faster after yielding compared with beams reinforced with BFRP bars.
(6) The ductility of the BFRP-SFRC beams was evaluated by the deformability-based approach. The deformability factor ranged from 6.54 to 10.68, which indicates that the beams had good ductility. For over-reinforced beams, the value of the deformability factor reduced as the reinforcement ratio increased from 1.15% to 1.65%. Therefore, it is suggested that the reinforcement ratio for the over-reinforced beams should be conservative.

## Figures and Tables

**Table 1:** Mix proportion.

W/C	Unit Weight (kg/m^3^)					
	Cement	Water	Sand	Gravel	Water Reducer	SF
0.31	529	164	706	1026	5.82	78.5

**Table 2:** Properties of SF.

Fiber Type	Diameter (mm)	Length (mm)	Aspect Ratio	Tensile Strength (MPa)	Elastic Modulus (GPa)
Hooked	0.55	35	65	1345	200

**Table 3:** Mechanical properties of reinforcing bars.

Bar Type	Diameter (mm)	Yield Strength (MPa)	Ultimate Strength (MPa)	Elastic Modulus (GPa)	Ultimate Strain
BFRP-1	12	-	1034.1	43.26	0.022
BFRP-2	14	-	1025.6	41.79	0.021
Steel-1	14	485	610	232.96	-
Steel-2	10	335	459.9	200.4	-
Steel-3	6	320	465	202	-

**Table 4:** Summary of flexural test results.

Beam	Crack Load (kN)	Maximum Load (kN)	Ultimate Mid-span Deflection (mm)	Maximum Crack Width (mm)		Failure Mode
				Ultimate Limit State	Serviceability Limit State	
B56	42.1	172.8	32.3	2.6	0.11	Tension failure
B77	44.0	244.0	35.7	3.5	0.33	Tension failure
B115	48.0	290.7	45.9	3.8	0.39	Compression failure
B165	52.3	358.1	48.2	4.0	0.31	Compression failure
S77	54.1	155.5	30.7	3.1	-	Tension failure

**Table 5:** Flexural bearing capacity degradation factors.

Beam	1Δ	2Δ	3Δ	4Δ	5Δ	6Δ	7Δ	8Δ	9Δ	Average
B56	90%	90%	91%	92%	89%	89%	-	-	-	90.2%
B77	88%	94%	94%	94%	94%	94%	86%	76%	-	90.0%
B115	91%	93%	93%	90%	92%	92%	90%	-	-	91.6%
B165	90%	95%	94%	93%	88%	92%	92%	87%	-	91.4%
S77	96%	86%	90%	90%	91%	90%	92%	92%	84%	90.1%

**Table 6:** Calculation results of deformability factor.

Specimen	M_ε_ = 0.001 (kN·m)	ϕ_ε_ = 0.001 (10^−5^/mm)	M_u_ (kN·m)	ϕ_u_ (10^−5^/mm)	C_s_	C_c_	J
B56	24.39	2.62	51.85	8.04	2.13	3.07	6.54
B77	31.96	2.36	73.28	9.44	2.29	3.99	9.13
B115	34.60	2.30	87.15	9.76	2.52	4.24	10.68
B165	39.52	2.23	107.43	8.68	2.71	3.89	10.54

## Data Availability

The data presented in this study are available on request from the corresponding author.

## References

[1] Mohamed H.M., Benmokrane B. (2014). Design and performance of reinforced concrete water chlorination tank totally reinforced with GFRP bars: Case study. J. Compos. Constr..

[2] Wang Z., Zhao X.L., Xian G., Wu G., Raman R.S., Al-Saadi S., Haque A. (2017). Long-term durability of basalt-and glass-fiber reinforced polymer (BFRP/GFRP) bars in seawater and sea sand concrete environment. Constr. Build. Mater..

[3] Hu S.W., Chen Q.Y., Gong N.N. (2018). Effect of acid corrosion on crack propagation of concrete beams. Sadhana.

[4] Xu G., Liu L., Bao H., Wang Q., Zhao J. (2017). Mechanical properties of steel corrosion products in reinforced concrete. Mater. Struct..

[5] Ahmed A., Al-Duais I., Riding K., Thomas M. (2018). Field evaluation of corrosion mitigation on reinforced concrete in marine exposure conditions. Constr. Build. Mater..

[6] Ashrafi H., Bazli M., Najafabadi E.P., Oskouei A.V. (2017). The effect of mechanical and thermal properties of FRP bars on their tensile performance under elevated temperatures. Constr. Build. Mater..

[7] Zomorodian M., Yang G., Belarbi A., Ayoub A. (2016). Cracking behavior and crack width predictions of FRP strengthened RC members under tension. Eng. Struct..

[8] Barris C., Torres L., Turon A., Baena M., Catalan A. (2009). An experimental study of the flexural behaviour of GFRP RC beams and comparison with prediction models. Compos. Struct..

[9] Wei B., Cao H.L., Song S.H. (2011). Degradation of basalt fibre and glass fibre/epoxy resin composites in seawater—ScienceDirect. Corros. Sci..

[10] Laoubi K., El-Salakawy E., Benmokrane B. (2006). Creep and durability of sand-coated glass FRP bars in concrete elements under freeze/thaw cycling and sustained loads. Cement. Concr. Comp..

[11] Almusallam T.H., Al-Salloum Y.A. (2005). Durability of GFRP rebars in concrete beams under sustained loads at severe environments. J. Compos. Mater..

[12] Abed F., Alhafiz A.R. (2019). Effect of basalt fibers on the flexural behavior of concrete beams reinforced with BFRP bars. Compos Struct..

[13] Fan X., Zhang M. (2016). Experimental study on flexural behaviour of inorganic polymer concrete beams reinforced with basalt rebar. Compos. Part B-Eng..

[14] Sim J., Park C., Moon D. (2005). Characteristics of basalt fiber as a strengthening material for concrete structures. Compos. Part B Eng..

[15] Herwig A., Motavalli M. (2012). Axial behavior of square reinforced concrete columns strengthened with lightweight concrete elements and unbonded GFRP wrapping. J. Compos. Constr..

[16] Rashid K. (2020). Cracking behavior of geopolymer concrete beams reinforced with steel and fiber reinforced polymer bars under flexural load. Compos. Part B-Eng..

[17] Cheng S. (2018). Flexural Behavior of High Strength Concrete Beams Reinforced with BFRP Bars and Steel Fiber. Ph.D. Thesis.

[18] Hollaway L.C. (2010). A review of the present and future utilisation of FRP composites in the civil infrastructure with reference to their important in-service properties. Constr. Build. Mater..

[19] Hassan M., Benmokrane B., Elsafty A., Fam A. (2016). Bond durability of basalt-fiber-reinforced-polymer (BFRP) bars embedded in concrete in aggressive environments. Compos. Part B-Eng..

[20] Elgabbas F., Ahmed E.A., Benmokrane B. (2016). Flexural behavior of concrete beams reinforced with ribbed basalt-FRP bars under static loads. J. Compos. Constr..

[21] Refai A.E., Abed F. (2015). Concrete contribution to shear strength of beams reinforced with basalt fiber-reinforced bars. J. Compos. Constr..

[22] Wu Z., Wang X., Wu G. (2012). Advancement of structural safety and sustainability with basalt fiber reinforced polymers. Proceedings of the 6th International Conference on FRP Composites in Civil Engineering (CICE).

[23] Shi J., Zhu H., Wu Z., Wu G. (2011). Durability of BFRP and hybrid FRP sheets under freeze-thaw cycling. Adv. Mater. Res..

[24] Elgabbas F., Vincent P., Ahmed E.A., Benmokrane B. (2016). Experimental testing of basalt-fiber-reinforced polymer bars in concrete beams. Compos. Part B-Eng..

[25] Wei B., Cao H., Song S. (2010). Environmental resistance and mechanical performance of basalt and glass fibers. Mater. Sci. Eng. A.

[26] Lee J.J., Song J., Kim H. (2014). Chemical stability of basalt fiber in alkaline solution. Fibres. Polym..

[27] (2006). Canadian Highway Bridge Design Code.

[28] (2015). Guide for the Design and Construction of Structural Concrete Reinforced with Fiber-Reinforced Polymer (FRP) Bars.

[29] Hua Y., Yin S., Feng L. (2020). Bearing behavior and serviceability evaluation of seawater sea-sand concrete beams reinforced with BFRP bars. Constr. Build. Mater..

[30] Ovitigala T., Ibrahim M.A., Issa M.A. (2016). Serviceability and Ultimate Load Behavior of Concrete Beams Reinforced with Basalt Fiber-Reinforced Polymer Bars. ACI Struct. J..

[31] Issa M.S., Metwally I.M., Elzeiny S.M. (2011). Influence of fibers on flexural behavior and ductility of concrete beams reinforced with GFRP rebars. Eng. Struct..

[32] Asfaour A. (2006). Flexural and shear capacities of concrete beams reinforced with GFRP bars. Constr. Build. Mater..

[33] Chellapandian M., Mani A., Prakash S.S. (2020). Effect of macro-synthetic structural fibers on the flexural behavior of concrete beams reinforced with different ratios of GFRP bars. Compos. Struct..

[34] Bischoff P.H., Gross S.P. (2011). Design approach for calculating deflection of FRP-reinforced concrete. J. Compos. Constr..

[35] Mousavi S.R., Esfahani M.R. (2013). Effective moment of inertia prediction of FRP-reinforced concrete beams based on experimental results. J. Compos. Constr..

[36] Zhu H., Cheng S., Gao D., Neaz S.M., Li C. (2018). Flexural behavior of partially fiber-reinforced high-strength concrete beams reinforced with FRP bars. Constr. Build. Mater..

[37] Yang J.M., Min K.H., Shin H.O., Yoon Y.S. (2012). Effect of steel and synthetic fibers on flexural behavior of high-strength concrete beams reinforced with FRP bars. Compos. Part B-Eng..

[38] Lee W.K., Jansen D.C., Berlin K.B., Cohen I.E. (2010). Flexural cracks in fiber-reinforced concrete beams with fiber-reinforced polymer reinforcing bars. ACI Struct. J..

[39] Gao D., Gu Z., Zhu H., Huang Y. (2020). Fatigue behavior assessment for steel fiber reinforced concrete beams through experiment and fatigue prediction model. Structures.

[40] Li C., Zhu H., Niu G., Cheng S., Gu Z., Yang L. (2022). Flexural behavior and a new model for flexural design of concrete beams hybridly reinforced by continuous FRP bars and discrete steel fibers. Structures.

[41] Mydin M.A.O. (2013). Engineering Performance of High Strength Concrete Containing Steel Fiber Reinforcement. http://anale-ing.uem.ro/2013/312.pdf.

[42] Shen D., Wen C., Zhu P., Li M., Ojha B., Li C. (2020). Bond behavior between basalt fiber-reinforced polymer bars and concrete under cyclic loading. Constr. Build. Mater..

[43] Banjara N.K., Ramanjaneyulu K. (2019). Investigations on behaviour of flexural deficient and CFRP strengthened reinforced concrete beams under static and fatigue loading. Constr. Build. Mater..

[44] Li Z., Qi Y., Teng J. (2020). Experimental investigation of prefabricated beam-to-column steel joints for precast concrete structures under cyclic loading. Eng. Struct..

[45] Yoo D.Y., Banthia N., Yoon Y.S. (2016). Predicting service deflection of ultra-high-performance fiber-reinforced concrete beams reinforced with GFRP bars. Compos. Part B-Eng..

[46] Naaman A.E., Jeong S.M. Structural ductility of concrete beams prestressed with FRP tendons. Proceedings of the Second International RILEM Symposium (FRPRCS-2): Non-Metallic (FRP) for Concrete Structures.

[47] Jaeger G.L., Tadros G., Mufti A.A., Halifax N.S. (1995). Balanced Section, Ductility and Deformability in Concrete with FRP Reinforcement.

[48] Wang H., Belarbi A. (2011). Ductility characteristics of fiber-reinforced-concrete beams reinforced with FRP rebars. Constr. Build. Mater..

